# A Sentinel in the Crosstalk Between the Nervous and Immune System: The (Immuno)-Proteasome

**DOI:** 10.3389/fimmu.2019.00628

**Published:** 2019-03-29

**Authors:** Fiona Limanaqi, Francesca Biagioni, Anderson Gaglione, Carla Letizia Busceti, Francesco Fornai

**Affiliations:** ^1^Human Anatomy, Department of Translational Research and New Technologies in Medicine and Surgery, University of Pisa, Pisa, Italy; ^2^I.R.C.C.S Neuromed, Pozzilli, Italy

**Keywords:** proteasome, immunoproteasome, T-cells, neuro-immunological synapse, cytokines, neurodegeneration, mTOR

## Abstract

The wealth of recent evidence about a bi-directional communication between nerve- and immune- cells revolutionized the traditional concept about the brain as an “immune-privileged” organ while opening novel avenues in the pathophysiology of CNS disorders. In fact, altered communication between the immune and nervous system is emerging as a common hallmark in neuro-developmental, neurodegenerative, and neuro-immunological diseases. At molecular level, the ubiquitin proteasome machinery operates as a sentinel at the crossroad between the immune system and brain. In fact, the standard proteasome and its alternative/inducible counterpart, the immunoproteasome, operate dynamically and coordinately in both nerve- and immune- cells to modulate neurotransmission, oxidative/inflammatory stress response, and immunity. When dysregulations of the proteasome system occur, altered amounts of standard- vs. immune-proteasome subtypes translate into altered communication between neurons, glia, and immune cells. This contributes to neuro-inflammatory pathology in a variety of neurological disorders encompassing Parkinson's, Alzheimer's, and Huntingtin's diseases, brain trauma, epilepsy, and Multiple Sclerosis. In the present review, we analyze those proteasome-dependent molecular interactions which sustain communication between neurons, glia, and brain circulating T-lymphocytes both in baseline and pathological conditions. The evidence here discussed converges in that upregulation of immunoproteasome to the detriment of the standard proteasome, is commonly implicated in the inflammatory- and immune- biology of neurodegeneration. These concepts may foster additional studies investigating the role of immunoproteasome as a potential target in neurodegenerative and neuro-immunological disorders.

## Introduction

In the last decades, wide evidence about a bi-directional communication between nerve- and immune- cells led to connect the two systems within the branch of neuro-immunology (1, 2). The functional connections between the immune and nervous system are based on common phylogenetic and embryological roots (3, 4), which are evident at both anatomical and molecular levels. Grossly, this occurs through (i) the sympathetic, mainly catecholamine, innervation of both primary and secondary lymphoid organs (5–8), and (ii) the recently discovered lymphatic pathways operating in the perivascular and meningeal spaces (9–13). Catecholamine released from sympathetic nerve terminals modulates immune activity through binding to neurotransmitter receptors, which are abundantly expressed on lymphoid cells (5–8). The amount and duration of released catecholamine, mostly dopamine (DA), dictates the stimulation/expression pattern of DA-receptors expressed on T-cells. This is seminal to activate specific intracellular cascades which in turn foster T-cell activation or suppression, T-cell differentiation toward effector vs. regulatory or memory cells, as well as migration of T-cells to non-lymphoid organs (8). At the same time, macroscopic convective fluxes of the glymphatic system enable the brain to drain the interstitial fluid (ISF) into the cerebrospinal fluid (CSF); CSF bearing soluble and cellular constituents is then drained into the bloodstream via arachnoid granulations and dural sinuses, and also directly into the deep cervical lymph nodes via dural lymphatic vessels (9–13). In this way, the clearance of potentially threatening interstitial solutes is achieved, and CNS-derived antigens (Ags) are drained to antigen presenting cells (APCs) in the choroid plexus, leptomeningeal spaces, and eventually, or even directly, in the deep cervical lymph nodes (14, 15). Within APCs, the ubiquitin proteasome (UP) and autophagy (ATG) machineries process endogenously- and exogenously- derived proteins into peptide determinants, which bind to major histocompatibility complex (MHC) molecules class I and II, respectively. CNS-derived Ags bound to MHC-I and -II are then exposed on the plasma membrane of APCs for presentation to CD8+ and CD4+ T-lymphocytes, respectively (16). Nonetheless, alternative pathways exist through which exogenously-derived Ags are cross-processed by the UP to combine with MHC-I and stimulate CD8+ T-cells (17); *vice versa*, endogenously-derived Ags (e.g., self- and viral peptides) can access MHC-II groove for presentation to CD4+ T-cells (18). Following associative binding of MHC molecules with T-cells receptors (TCR), presentation of CNS-derived Ags fosters activation of T-cells in periphery, while mounting CNS-directed adaptive immune responses, which may have either beneficial or detrimental effects (14, 15, 19, 20). Peripherally activated T-cells can enter the brain parenchyma by crossing all CNS barriers including the blood-CSF, the blood-leptomeningeal, and the blood-brain barrier (19, 21). Along these barriers primed CD4+ and/or CD8+ T-cells encounter APCs which expose the cognate Ag complexed with MHC-II and/or MHC-I. In the presence of specific signals (e.g., co-stimulatory molecules, adhesion ligands and inflammatory cytokine mediators), re-activation of CD4+ and CD8+ T-cells by APCs leads to the recruitment of their effector machinery to produce pro-inflammatory cytokine release and cytotoxicity, respectively (14, 15, 19–22). The foremost professional APCs which foster re-activation of T-cells in the CNS are dendritic cells (DCs) and macrophages in the CSF, perivascular space and choroid plexus stroma; nonetheless, choroid epithelial cells and endothelial cells of the CNS microvasculature also behave as CNS-resident APCs, thus providing a pathway for T-cell re-activation and infiltration in the brain (23, 24). It is remarkable that once in the CNS parenchyma, T-cells interact with, and may also target glia and neurons, which indeed are able to operate as APCs (15, 25). This is magnified under oxidative/pro-inflammatory conditions, where glia and neurons readily upregulate their ability to process, load and present Ags via MHC-I/-II and MHC-I molecules, respectively (15, 25). In this novel scenario, neurotransmitters and classic immune-related molecules co-operate at the level of a hybrid junction, the “neuro-immunological synapse,” where they adopt a common language to modulate both synaptic plasticity and neuro-immunity (26, 27). These findings have revolutionized the traditional concept of the brain as an “immune-privileged” organ while opening novel clues in the pathophysiology of CNS disorders (14, 28). In fact, defective or inappropriate communication between the immune and nervous system is emerging as a common hallmark in a number of etiologically different CNS diseases including neuro-developmental, neurodegenerative and neuro-immunological disorders (28). At molecular level, the UP represents an evolutionary preserved catalytic machinery operating at the cross-road between synaptic and immune activity (29–32). Dysregulations of the UP characterize a variety of neurological disorders where immune alterations occur, such Multiple Sclerosis (MS) and neuro-infectious diseases, but also classic neurodegenerative disorders such as Parkinson's, Alzheimer's, and Huntingtin's diseases (PD, AD, HD), epilepsy, brain stroke, and drug abuse (33–42). As such, the contribute of UP in the context of inflammatory- and immune-related biology of CNS disorders has been increasingly investigated (43, 44). The present mini-review analyzes those UP-related molecular mechanisms underpinning the shift from baseline neuro-immune surveillance to inflammatory and auto-immune neuropathology.

## Proteasomes in Immune Cells and Implications for Brain Functions

Proteasomes are ubiquitous multi-subunit proteases which ensure cell homeostasis. Such a task is achieved by removing unfolded, misfolded, oxidized, or disordered proteins to prevent their accumulation, aggregation, and spreading (45, 46). As actors of protein degradation, proteasomes regulate most cell functions encompassing cell cycle and division, cell differentiation and development, oxidative/inflammatory stress, and immune response. To optimize these different tasks according to specific cell demands, evolution has preserved alternative subtypes of proteasomes, which despite overlapping in structure and functions, differ for catalytic subunits and substrate specificity (29–32, 45–47). In the present review, we focus on two major proteasome isoforms, namely the standard 26S proteasome (SP) and its alternative/inducible counterpart, the immunoproteasome (IP), which operate dynamically and coordinately in nerve, glial, and immune cells to modulate neurotransmission, oxidative/inflammatory stress response and immunity. In the present section, we discuss the mechanisms through which SP and IP tune the repertoire of brain-circulating T-lymphocytes. Circulation of T-cells in the CNS occurs since the early development to guarantee both immune-surveillance and synaptic plasticity (48–50). On the other hand, alterations in CNS-circulating T-cell populations are emerging as a common signature in both classic and autoimmune degenerative disorders such as PD, AD, and MS (51).

### Standard Proteasome Bridging Neurotransmission With Lymphocyte Activity

The SP is ubiquitously expressed in non-immune cells including neurons, where it operates in the nucleus, cell body, and synapses to modulate oxidative stress, gene transcription, neurotransmitter release and synaptic plasticity (31, 52, 53). This is validated in a plethora of experimental models where SP inhibition profoundly alters neurotransmitter release and the expression of neurotransmitter receptors while producing ubiquitinated protein-aggregates, which recapitulate neurodegeneration (54–58). As a support to these findings, SP dysfunctions in human CNS disorders are bound to early synaptic alterations and/or protein aggregation (36–38, 59–61). Although detailing the mechanisms of SP in synaptic plasticity is beyond the aim of this brief review, we wish to mention that SP may modulate immune activity by modulating in turn, neurotransmitter release. For instance, SP modulates dopamine (DA) release (55–57), which is seminal for differentiation, maturation, selection, trafficking, and migration of T-lymphocytes (7, 8, 62–65). This occurs through the stimulation of DA receptors (D1–D5), which are all expressed on T-cells. Just like it occurs for neurons, the magnitude and duration of DA release are seminal to dictate the intracellular cascades placed downstream to DA receptors (DRs) in T-cells (8, 61–64). For instance, abnormal stimulation of D1/D5-DRs increases cAMP levels to inhibit activation of cytotoxic CD8+ T-lymphocytes (CTLs); again, it induces polarization of naïve CD4+ T cells toward T helper type17 phenotype (Th17) while suppressing differentiation and activity of T regulatory cells. On the other hand, stimulation of D3-DRs controls T-cell adhesion and migration and induces differentiation of naïve CD8+ T-cells into CTLs; again, it induces polarization of naïve CD4+ cells toward Th1 phenotype. Thus, SP-dependent surveillance of DA release and stimulation of DA-receptors at the level of the neuro-immunological synapse, in cooperation with CNS-derived Ag presentation, plays an active role in determining T-cells fate and activity, as well as their chemotactic migration and homing to the CNS. Emerging evidence indicates an association between T-cell-related pro-inflammatory and autoimmune mechanisms underlying neuropathology with abnormal DA levels and deregulation of DA receptors expressed on T-cells (64–69). It is remarkable that this occurs CNS disorders such as MS, PD, and stroke, where SP is impaired while its immune-related counterpart (the IP) is upregulated (34).

Besides the effects in lymphoid organs, DA release also modulates T-cells activity directly in the brain, including activation or suppression of naïve T-cells [*for a review* (8)]. In fact, despite the consensus view that only activated T-cells can migrate into the brain, a number of studies also revealed an unexpected ability of naïve CD4+ and CD8+ T cells to infiltrate the brain parenchyma (70–76). This is magnified during pro-inflammatory conditions, which enhance naïve T-cell recruitment in the CNS, while fostering their activation once they encounter the specific Ag (71, 74, 76–78). This was shown to occur upon interaction of naïve CD4+ and/or CD8+ T-cells with either activated microglia or oligodendrocytes [ODCs, (71, 74, 76, 77)]. However, the specific molecular mechanisms and functional significance underlying this phenomenon still remain to be elucidated. Recent *in vitro* studies demonstrated that exogenous administration of DA precursors to neurons which are co-cultured with activated CD8+ T-cells is sufficient to induce cognate Ag presentation via MHC-I and subsequent CTL-mediated neuronal death (79). Due to its intrinsic oxidative potential, DA is considered the primary candidate fostering SP disassembly and subsequent IP upregulation (40). This is supported by the effects of DA in enhancing neuronal Ag presentation via MHC-I (79), which is indeed the main task of IP (section Immunoproteasome in Constitutive and Adaptive Immunity). Thus, in a scenario in which dysfunctional SP alters DA release, the upregulation of IP renders neurons competent APCs for presentation to CD8+ T-cells; at the same time, abnormal stimulation of DA receptors (for instance D3-DRs) on CD8+ T-cells triggers metabolic downstream cascades which add on the recruitment of cytotoxic T cell effector machinery.

### Immunoproteasome in Constitutive and Adaptive Immunity

**Figure 1:**
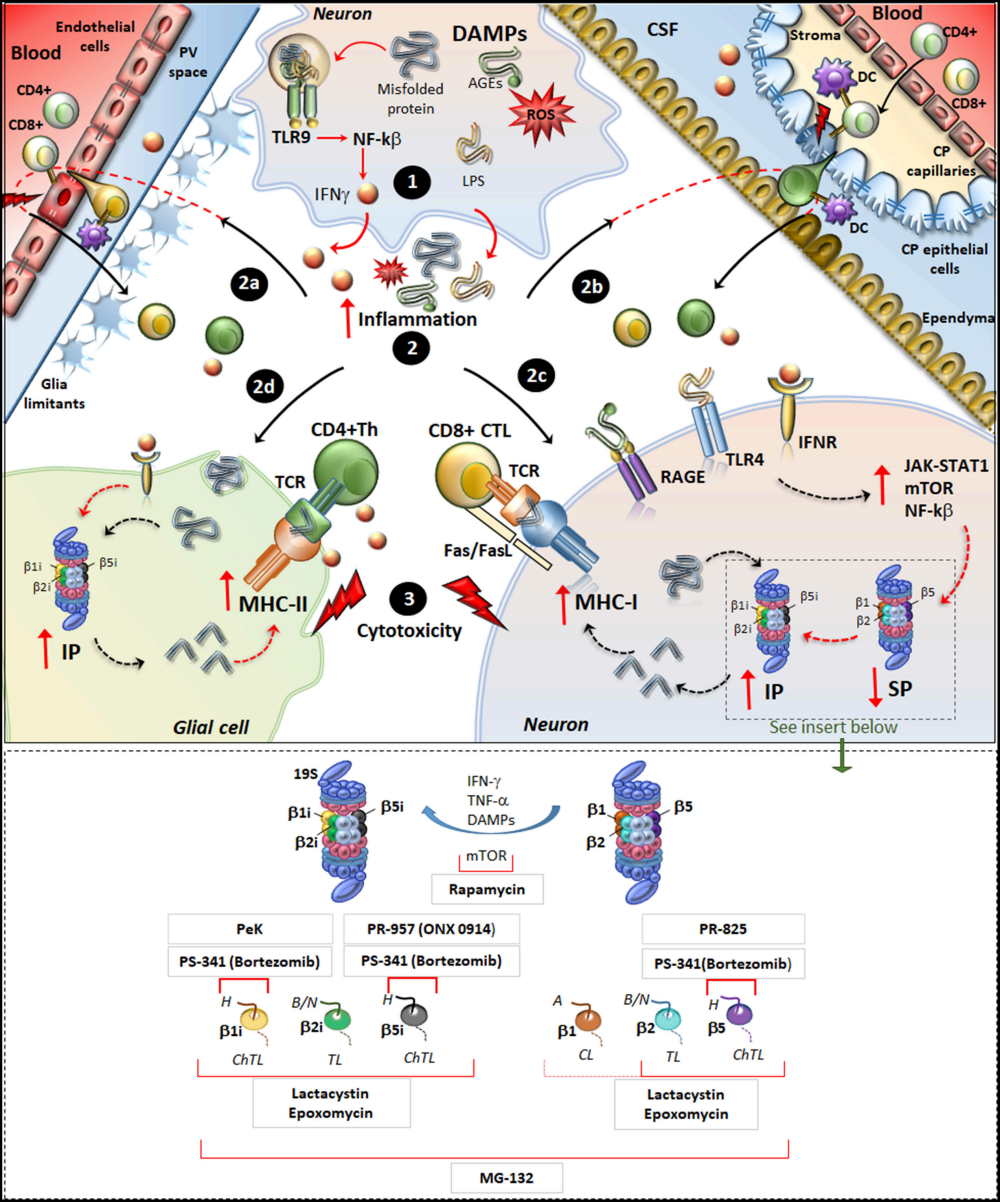
Molecular mechanisms underlying IP induction in neurons and glia in neurodegenerative disorders. (Upper panel) Within neurons, an oxidative/inflammatory challenge or the presence of misfolded proteins leads to the production of DAMPs such as ROS, LPS, and AGEs. DAMPs bind to TLR9 to activate NF-kb and produce pro-inflammatory cytokines (1). DAMPs and misfolded/oxidized proteins and cytokines are then released extracellularly, which triggers an inflammatory reaction within the brain parenchyma (2). This fosters the recruitment of peripherally primed T-cells which are reactivated by APCs along the blood-brain barrier (2a) and blood-CFS barrier (2b), including DCs in the perivascular space (PV), in the choroid plexus (CP) stroma and CSF, as well as CP epithelial cells and endothelial cells of the brain-blood-barrier. In this way auto-reactive CD4+ T cells (green) and CD8+ T-cells (yellowish) recruit their effector machineries to damage CNS barriers (flashlights) and infiltrate the brain parenchyma. At the same time, misfolded/oxidized proteins, DAMPs and IFNs spread throughout the brain parenchyma and they bind to their receptors IFNr, RAGEs and TLR4 which are expressed in glia and neurons (2c, 2d). These activate common intracellular pathways namely JAK/STAT, NF-kβ, and mTOR, which downregulate/disassembly SP to foster induction and *de-novo* synthesis of IP. Thus, IP produces Ag peptides which bind to MHC-I molecules in neurons (2c) or even to MHC-II in glia (2d). MHC-antigen complexes are then transported to the cell surface to be presented to auto-reactive CD8+ CTLs and CD4+ Th lymphocytes, which trigger cytotoxicity and cytokine-mediated damage in neurons and glia (3). Figure Insert. Schematic overview of the mechanism of action of various IP/SP inhibitors listed in Table 1. On the right, the SP with its subunits β1, β2, and β5 which possess caspase-like (CL), trypsin-like (TL) and chymotrypsin-like (ChTL) activity, respectively. Following inflammatory/oxidative stimuli (IFN-γ, TNF-α, or DAMPs release), SP subunits are replaced with IP subunits and de-novo synthesis of IP occurs. On the left, IP with its subunits β1i, β2i and β5i which possess ChTL, TL, and ChTL activity, respectively. *Rapamycin*, mTOR inhibitor, reduces the synthesis of IP subunits and enhances P26S-dependent protein degradation; *PeK (Peptidyl epoxyketone)*, selective epoxyketone of inhibitor of β1i; *PS-341 (Bortezomib)*, reversible dipeptide boronate inhibitor of SP and IP with high affinity for β5, β5i, and β1i; *PR-957 (also known as ONX 0914)*, irreversible β5i -selective epoxyketone inhibitor; *PR-825*, irreversible β5-specific inhibitor; *Epoxomycin*, irreversible and selective inhibitor of both SP and IP with high affinity for β2, β5, β1i, β2i, β5i; *Lactacystin, similar to Epoxomycin; MG-132*, nonspecific inhibitor of all β subunits of the 20S core particles within both SP and IP. H, hydrophobic; B/N, basic/neutral; A, acidic substrates.

The IP is an alternative, cytokine-inducible form of the SP, which is mostly involved in inflammatory and immune response (80). All immune cells, including professional APCs (e.g., DCs) and lymphocytes, possess almost exclusively IP. Within APCs the IP generates defined T-cell epitopes which bind to MHC-I molecules (81–83). In detail, IP cleaves either endogenous or exogenous proteins to generate Ag peptides, which are firstly complexed to MHC-I in the endoplasmic reticulum and then exposed on the plasma membrane of APCs, for either direct or cross-presentation to CD8+ T-lymphocytes. This is accomplished at a higher rate and with greater efficacy by IP since it owns a selective enhancement of chymotrypsin-like activity and unique structural features compared with SP. In detail, within IP, β_1_, β_2_, and β_5_ subunits of the SP-20S catalytic core are replaced with β_1_i or low molecular mass protein 2 (LMP2), β_2_i or multi-catalytic endopeptidase complex subunit-1 (MECL-1) and β_5_i or LMP7, respectively (81–83). LMP2 possesses chymotrypsin-like activity contrarily to the standard β_1_ counterpart which possesses caspase-like activity. Moreover, LMP7 which possesses chymotrypsin activity similarly to the β5 subunit of SP, has a unique hydrophilic architecture which surrounds the LMP7-oxyanion hole (82). This facilitates the generation of peptides with C-terminal hydrophobic and basic amino acids, which better fit into the groove of MHC-I molecules (82, 84, 85). In this way, peptides bound to MHC-I are exposed extracellularly on the plasma membrane of DCs to be recognized by CD8+ T-cells as modified compared with “self” Ags. This is seminal to avoid auto-immunity while mounting T-cell mediated adaptive immune responses for the removal of pathogen-infected cells (86). Besides Ag presentation, the IP also operates within naïve T-cells to modulate metabolic cascades which orchestrate their differentiation and function (87). For instance, IP governs CD4+ T-cell differentiation toward T helper (Th1 and Th17) vs. T regulatory cell lineage (88). Likewise, IP regulates CD8+ T lymphocyte metabolism and differentiation toward memory vs. effector cells (89). IP also sustains the maturation process of stimulated DCs from an Ag-receptive state to a state of optimal stimulation of T-cells (90). Again, specialized and classic subtypes of IPs operate in thymic DCs, where together with SP, they regulate T-cell proliferation along with positive and negative T-cell selection (91, 92). SP-derived pool of peptides differs from that produced by IP degradation, and this is critical to avoid generation of auto-reactive T-cells. In this way, SP and IP coordinately shape the repertoire of immunocompetent T-cells, which are released in the bloodstream to reach secondary lymphoid organs and subsequently the brain via the CSF. Immune adaptation of the UP is a tightly regulated and transient response, which allows cells to rapidly switch back to SP once IP function is no longer required (93). In fact, production of IP in response to pro-inflammatory cytokines such as IFN-γ is four times faster than SP. This allows cells to quickly expand the peptides repertoire which is needed to aid immune defense in a challenged organism. Likewise, IP turnover is definitely faster compared with SP in order to avoid persistent immune activation (93). The transient induction of IP is seminal to protect the brain against microbial infections. In fact, IP inhibition may increase the susceptibility to either viral, fungal or bacterial neuro-infections (89, 94, 95). This correlates with profound alterations in T-cells differentiation and function along with altered cytokine release (89, 94–97). However, under persistent pro-inflammatory and/or oxidative stimuli, the balanced tuning between SP and IP fails to occur leading to an abnormal prevalence of IP over SP. In turn, abnormal IP upregulation enhances generation and MHC-I-dependent presentation of CNS-derived Ags within DCs while producing metabolic/transcriptional changes within both DCs and T-cells. These effects eventually synergize to produce CNS-directed auto-immune reactions. In the light of these findings, IP and/or SP inhibitors have been tested as a potential therapeutic strategy in CNS auto-immune disorders such as experimental autoimmune encephalomyelitis (EAE), and also in neurological disorders, which etiologically are not bound to auto-immunity [(98–127); Table 1; insert of Figure 1 for details]. Since IP operates in neurons and glia in addition to classic DCs, in the next paragraph we discuss evidence centered on IP expression within the CNS and its contribution to pro-inflammatory and auto-immune neuronal damage.

**Table 1:** Mechanisms of action of IP and/or SP inhibitors and their reference to IP and SP status in specific CNS disorders.

**Mechanism of action of proteasome inhibitors tested in CNS disorders** ***PR-957** (also known as ONX 0914)*: irreversible β5i -selective epoxyketone inhibitor (98, 99) ***PR-825***: irreversible β5-specific inhibitor (99, 100) ***PeK** (Peptidyl epoxyketone)*: β1i-selective epoxyketone inhibitor (101) ***PS-341** (Bortezomib)*: reversible dipeptide boronate inhibitor of SP and IP with high affinity for subunits (β5, β5i and β1i) with chymotrypsin-like activity (101, 102) ***PS-519** (Lactacystin-like compound)*: irreversible inhibitor of both SP and IP with higher affinity for chymotrypsin- and trypsin-like activity (β2, β5, β1i, β2i, β5i) (54, 57, 103) ***Epoxomycin***: irreversible and selective inhibitor of both SP and IP with high affinity for chymotrypsin-like and trypsin-like activity (β2, β5, β1i, β2i, β5i) (54) ***MG-132:*** nonspecific inhibitor of all β subunits of the 20S core particles within both SP and IP (101, 104) ***Rapamycin***: mTOR inhibitor, reduces the synthesis of IP subunits (105, 106) and enhances P26S-dependent protein degradation (107)		
**CNS disease**	**IP and SP status ↑ increased; ↔unchanged; ↓decreased**	**Effects of IP and/or SP inhibitors tested in experimental models**
MS	Humans	
	↓ catalytic activities and ↔ protein levels of β1, β2, and β5 in post-mortem brain samples (gray and white matter) from MS patients (37)	
	↑ immune-histochemical reactivity for β1i in the cortex and white matter of post-mortem CNS samples from MS but not young controls. In MS brain specimens β1i is detected in both glial cells and neurons and it co-localizes with plaques (108)	
	Experimental models	
	↑β1i and β5i in the brains of Myelin Basic Peptide (MPB)-EAE mice compared, with β1i being dominantly expressed in ODCs and β5i in brain-infiltrating lymphocytes (101)	ONX 0914 ameliorates Myelin Olygodendrocyte Glycoprotein (MOG)-EAE and Proteolipid protein (PLP)-EAE by inhibiting naïve CD4+ T cells differentiation toward Th17/1 phenotype in lymph nodes and by reducing infiltration of cytokine-producing CD4+ T cells in the brain and spinal cord (98)
	*Ex vivo*, β1i and β5i from the brain of EAE mice produce a release of immunogenic MBP peptides which is 10-fold higher compared with control mice possessing low levels of IP. *Ex vivo*, IP-dependent release of MBP from EAE mice induces CTL-mediated targeting of ODCs (101)	PEk inhibits chymotrypsin-like activity in MBP-EAE mice brains by 70% and ameliorates demyelination pathology at a higher rate compared with PS-341 (101)
	↑ amount and activities of β1i, β2i, and β5 in glia and neurons of MOG-EAE rats (102)	PEk, PS-341, and MG-132 all efficiently inhibit the release of immunogenic myelin basic protein peptides by proteasomes from MBP-EAE mice brains *ex vivo* (101)
	↑ overall peptidase proteasome activity during the acute phase of EAE correlates with ↑levels of β1i and β5i in neurons and glia of MOG-EAE mice (109, 110)	
	↓ overall peptidase proteasome activity during the chronic phase of EAE correlates ↓ levels of β1, β2 in neurons and glia of MOG-EAE mice (109, 110)	Bortezomib significantly reduces clinical EAE score and disease progression in MOG-EAE mice by lowering the number of IFN-γ and IL-17 producing cells from spleens of EAE mice and NF-κB activity in the spleen and CNS of MOG-EAE mice compared with vehicle-treated controls (102)
		Bortezomib improves the neurological outcome and reduces the cumulative clinical score in MOG-EAE rats (102)
		PS-519 reduces clinical score and relapses in PEP-Relapsing EAE mice, by ameliorating NF-κB-mediated inflammatory and demyelinating histopathology in the spinal cord, and by reducing Th1 responses in the spleen and lymph nodes from PEP-Relapsing-EAE mice compared with vehicle-treated controls (103)
AD	Humans	
	↓overall chymotrypsin and caspase-like activities and ↔ protein levels of β subunits in AD post-mortem brain samples (59, 111)	
	↓gene expression of β5 and ↑gene and protein levels of β5i and β1i in hippocampi of post-mortem AD brains (112, 113)	
	↑activities of β5i, β1i, β2i in hippocampi of post-mortem AD brains correlating with tau pathology (112)	
	Age-related ↑of β5i and β1i in human brain tissues (114)	
	↑β1i and ↓β1 levels in AD affected brain regions (hippocampus) from post-mortem human samples compared with non-affected brain regions from AD patients and age-matched controls (114)	
	↑ immune-reactivity for β2i and β5i in neurons and mostly in glial cells in the hippocampi of post-mortem AD brains (113)	
	Experimental models	
	Age-related ↑β5i and β1i, and ↓β5 and β1 in rats' hippocampi. LPS injections reproduces these features also in young rats, while spatial memory training reverses IP/SP ratio (115)	ONX-0914 exposure reduces pro-inflammatory signaling in *ex vivo* microglia isolated from AD mice, while PR-825 does not produce significant effects (112)
	Age-dependent ↑gene expression and protein levels of β5i and β1i in neurons and glial cells surrounding Aβ plaques in AD mice (112)	β5i knockdown in AD mice models improves amyloid-beta (Aβ)-associated cognitive deficits by altering cytokine response in microglia but does not affect Aβ levels (117)
	↑ activities of β5i, β1i, β2i, β2, and ↔ activities of β1 and β5 subunits in AD mice compared with age-matched controls (112)	Lactacystin administration following LPS injections induces neuronal accumulation of ubiquitinated proteins, expression of pro-apoptotic markers and neurodegeneration in rats (118)
	↓β5 and ↑β1i and β2i levels, and ↑trypsin-like activity in AD mice (116)	
	↑gene expression and protein levels of β5i and β1i, correlates with aging and Aβ-pathology in AD mice (117)	
PD and DLB	Humans	
	↑β5i levels and ↑chymotrypsin activity in neurons and glial cells of post-mortem brains from patients with PD and Dementia with Lewy Bodies (DLB, 119)	ONX-0914 exposure results in greater intracellular accumulation of alpha-synuclein *in vitro* (119)
		ONX-0914 administration exacerbates 6-OHDA-induced neurotoxicity *in vitro* and *in vivo* (120)
	Experimental models	
	↑β5i levels in 6-OHDA mice models of PD (120)	Lactacystin or epoxomycin microinfusions within the Substantia Nigra of rats induce nigrostriatal toxicity which reproduce PD neuropathology (54)
		Lactacystin injected into the medial forebrain bundle in minipigs provides a model of PD with reduced DA neurotransmission, catecholamine neuron loss, microglial activation and behavioral deficits (57)
HD	Humans	
	↓overall chymotrypsin-like activity in the brains and fibroblast of post-mortem HD samples (60)	
	↑β1i and β5i and ↓β1 and β5 levels in the degenerating and aggregate-containing neurons of post-mortem HD brains (121)	Lactacystin increases the accumulation of mutant HD exon-1 protein aggregates *in vitro* (122, 123)
	Experimental models	
	↑β1i and β5i levels and ↑chymotrypsin-like activity in neurons and glia within the cortex and striatum of HD mice, with β1i localizing mainly in degenerating neurons (121)	
Ischemic stroke	Humans	
	↑β1i, β2i, and β5i levels in plasma of ischemic stroke patients and predicts early hemorrhagic transformation in acute ischemic stroke (124)	
	Experimental models	
	↑β1i and β5i within neurons of the parietal cortex and hippocampus in a mice model of transient focal cerebral ischemia (125)	β1i knockdown or MG-132 administration prior to MCAO ameliorate brain infraction volume in rats by reducing pro-inflammatory cytokines production and glial cells activation, with infraction volumes being smaller in β1i-silenced compared with MG-132 treated mice (104)
	↑β1i and β5i in the ischemic cerebral cortex and striatum of rats with middle cerebral artery occlusion (MCAO) (104)	
Epilepsy	Humans	
	↑β1i and β5i in neurons and glia in surgically resected temporal lobe epilepsy (TLE) hippocampi and in focal cortical dysplasia (126)	Rapamycin downregulates expression of IP subunits β1i and β5i in glial cell cultures from patients with malformations of cortical development (MCD, 38)
	↑β1i, β5i, β1, and β5 levels in neurons and glia from patients with malformations of cortical development (38) and drug-resistant TLE (127)	Rapamycin ameliorates post-status epilepticus (SE) in rat models of TLE by downregulating β1i and β5i in neurons and glia. Rapamycin downregulates β1i and β5i in glial cell cultures from patients with drug-resistant TLE (127)
	Experimental models	
	↑β5i gene expression and protein levels and ↔ levels of SP subunits in the hippocampal/entorhinal cortex from rat models of 4-aminopyridine-induced chronic epilepsy (100)	ONX-0914 prevents the onset of seizure-like events (SLEs) in hippocampal/entorhinal cortex slices from chronic epileptic rats, and such an effect is not reproduced by PR-825 (100)
	↑β1i and β5i levels correlate with seizure frequency in a rat model of TLE (127)	

## The Role of Proteasomes in Neurons and Glia

In neurons and glial cells, IP is generally induced by the pro-inflammatory cytokines IFNγ and TNFα, and by oxidative stress (42, 80, 82, 128). In these challenging conditions, SP disassembles to produce IP, which it is suggested to boost protein degradation and cope with protein overload (42, 119, 129, 130). Since IP owns enhanced catalytic activity, it produces immunogenic polypeptides from both microbial- and oxidized/aggregated-proteins. In fact, IP degrades aggregation-prone proteins such as alpha-synuclein with a similar or even higher rate and efficacy compared with SP (130, 131). Remarkably, IP cleaves alpha-synuclein specifically within immunogenic sites (119, 132), thus providing an oxidation-linked rationale for its Ag processing role in neuro-immune surveillance (32). This may explain why neurons and glia express low amounts of IP also in the absence of cytokine stimulation, which suggests a homeostatic role of IP in the CNS (133). One function consists in maintaining the expression of MHC-I molecules within specific neuronal populations and glia throughout the brain and spinal cord (133–135). The expression of MHC-I in the CNS extends beyond a classic Ag-presenting role. In fact, neuronal expression of MHC-I is bound to early neuronal development, axonal regeneration, synaptic plasticity, reward and memory (25, 136–138). Nonetheless, IP-dependent Ag processing and subsequent MHC-I-dependent Ag presentation to CD8+ T cells enable neurons and glia to behave just like professional APCs do. Thus, following vicious cycles of inflammatory/oxidative stress in the CNS, a persistent increase of IP to the detriment of SP may render neurons and glia susceptible to auto-immune damage.

### Molecular Mechanisms Bridging Immunoproteasome With Immune Alterations and Neurodegeneration

The IP is significantly up-regulated in glia and neurons, in both patients and experimental models of HD (121, 139), AD (112–117), PD (119, 120), MS (41, 98–103, 108–110), ALS (134, 140), neurotrauma (129), ischemic stroke (104, 124, 125), and epilepsy (38, 100, 126, 127). In the context of PD, the induction of IP within glia and DA neurons was recently related to alpha-synuclein degradation and subsequent generation of self-Ag peptides for T-cell presentation by MHC-I (79, 119, 132). Since DA neurons of the Substantia Nigra (SN) possess an enhanced sensitivity to MHC-I upregulation, their susceptibility in PD may be related to CTL-mediated injury and death (79, 132). This hypothesis was tested by *in vitro* experiments showing that stem cell-derived DA neurons as well as murine primary catecholamine neurons can internalize, process and load Ags onto MHC-I just like professional APCs do (79). In detail, neuronal upregulation of Ag-loaded MHC-I can be induced by either microglial activation and subsequent IFN-γ release, or by administration of DA precursors even in the absence of microglia or exogenously administered IFN-γ. In the presence of activated CD8+ T-cells, the cognate Ag/MHC-I complex exposed on the neuronal plasma membrane induces proliferation of CD8+ T-cells, and most remarkably, it is sufficient to trigger CTL-mediated neuronal death via Fas/Fas ligand and perforin/granzyme pathways (79). Contrariwise, inflammatory-challenged neurons have no effects upon CD4+ T-cells, which specifically recognize MHC-II-bound Ags. This is in line with the lack of MHC-II expression in neurons either in baseline or inflammatory conditions. Nonetheless, Ag-peptides derived from alpha-synuclein degradation can presented via MHC-II molecules by glial cells for re-activation of CD4+ Th cells (119, 132). Thus, IP-dependent generation of Ag-peptides from alpha-synuclein may produce both pro-inflammatory and cytotoxic T-cell-mediated effects converging on DA neurons in PD. Despite being apparently detrimental, a balanced perspective emerges from experimental studies indicating a neuroprotective role for IP induction. In fact, the parkinsonian neurotoxin 6-hydroxydopamine (6-OHDA) increases IP and MHC-I expression in DA neurons *in vitro* and *in vivo* while IP inhibition exacerbates instead of preventing 6-OHDA-induced neurotoxicity (120). This suggests that in response to oxidative and inflammatory stimuli which foster protein-aggregation, transient induction of IP may compensate for SP downregulation to maintain cell-proteostasis. This is in line with studies on HD, showing that IP co-localizes with ubiquitinated aggregates in neurons from human and mouse brains (121). Noteworthy, a marked increase in IP induction takes place only at advanced stages of HD, when substantial proteinopathy develops along with SP downregulation. Subsequent studies demonstrated that protein-misfolding needs to synergize with pro-inflammatory cytokines in order to reproduce IP upregulation of HD brains (139). These results confirm that IP induction follows neuro-inflammation, which develops during protein aggregation. This is reproduced in experimental models of AD (117), ALS (134, 140), neurotrauma (129), ischemic stroke (104), epilepsy (100, 127), and MS (101), where the onset of inflammation accelerates IP expression and neuropathology. In neurodegenerative disorders, overlapping molecular mechanisms operate to foster neuro-inflammation and IP induction in either neurons or glia. For instance, misfolded or oxidized substrates may *per se* trigger inflammation through the release of danger-associated molecular pattern molecules (DAMPs) (141). Within neurons or glia, DAMPs bind to Toll-like receptor 9 (TLR-9) expressed in endosomes, which activates Nf-Kβ to foster the production of inflammatory cytokines including IFNγ (Figure 1). The inflammatory milieu promotes the recruitment, re-activation and infiltration of auto-reactive T-cells in the CNS parenchyma. At the same time, IFNγ induces upregulation of IP either locally or within neighboring cells, via autocrine or paracrine mechanisms. Induction of IP also occurs following binding of DAMPs to Toll-like receptor 4 (TLR4) as well as binding of advanced glycated end products (AGEs) to their receptors [RAGEs, (100, 118, 142, 143)]. Similarly to what occurs for IFNs receptors, activation of TLR4 and RAGEs is coupled to intracellular signaling cascades, which induce IP while downregulating SP. These consist of activation of JAK-STAT1, Nf-Kβ, and mTORC1 pathways, which trigger production of pro-inflammatory cytokines, replacement of SP with IP subunits and *de novo* synthesis of IP subunits (33, 100, 105, 106, 142, 143). In this way, IP upregulation leads to overproduction of neuronal and/or glial Ags co-expressed with MHC-I molecules to activate CD8+ CTLs. In glial cells, IP may also cross-process Ags which bind on MHC-II molecules to prime CD4+ Th lymphocytes and fuel production of pro-inflammatory cytokines (119, 132) (Figure 1). This eventual IP-related mechanism is reminiscent of what occurs in EAE, though IP induction within oligodendrocytes (ODCs) following IFNγ-mediated inflammatory reaction, seems be primarily involved in auto-immune demyelination rather than representing a compensatory response to proteinopathy as it occurs in neurodegenerative disorders (101). In fact, the specific up-regulation of the IP subunit LMP2 within ODCs leads to efficient production of myelin basic peptides (MBP) recognizable by CTLs, which may occur in the absence of, or at least prior to MBP cross-presentation to CD4+ Th cells (101). As a support to these findings, an *LMP2* polymorphism, which alters production of MBP epitopes presented on MHC-I, associates with a reduced risk to develop MS in humans (108). At the same time, increased expression of LMP7 specifically in CD4+ CNS-infiltrating lymphocytes may facilitate Th17- and/or Th1-mediated damage in the CNS by stimulating their survival and proliferative capacity (88, 101, 108). The upregulation of IP in glial cells as well as in peripheral and CNS-circulating T-lymphocytes, joint to the beneficial effects observed upon selective IP inhibition in EAE, suggest that IP is mechanistically involved in the autoimmune nature of MS (41, 98, 101).

## Conclusions and Clinical Implications

The evidence here reviewed converges that changes in UP β-subunit composition are largely responsible for the fluctuations in UP activities, which were described during the progression from inflammatory to neurodegenerative stages. Thus, characterizing UP subunit composition and IP/SP ratio appears seminal, since enzymatic assays do not permit to establish the molecular origin of UP activities. While some mechanisms underlying the over-expression of IP are emerging, those underlying changes in the expression of SP in various CNS disorders still remain to be fully established. Moreover, SP status and the IP/SP ratio varies not only among different CNS disorders, but also among various disease stages. For instance, beneficial effects in some EAE models are observed also following inhibition of both IP and SP subunits, while in classic neurodegenerative disorders SP inhibition appears detrimental (Table 1). In any case, IP upregulation occurs independently of disease etiology following oxidative/inflammatory reactions in the CNS. Again, the functional significance of IP induction differs between MS compared with classic neurodegenerative disorders, which is likely to underlie their different etiologies. In neurodegenerative disorders, upregulation of IP occurs as a compensatory response to cope with jeopardizing inflammatory conditions, which develop during proteinopathy (121, 139). In fact, selective IP inhibitors do not substantially modify the amount of Aβ despite ameliorating inflammation and cognitive abilities in AD models (117). Likewise, selective inhibition of IP does not protect DA neurons from 6-OHDA neurotoxicity (120). Thus, neuro-inflammatory and autoimmune reactions in these disorders may relate to concomitant SP dysfunction, which is further sustained by IP upregulation. This calls for a careful evaluation of SP status and activity in experimental approaches aimed at inhibiting the IP (see insert of Figure 1). For instance, targeting common pathways through which IP operates in the CNS may foster the naturally occurring switch from IP to SP. This is the case of mTOR inhibitors, which downregulate IP while counteracting protein aggregation and inflammation (38, 143–145). In addition to the effects upon SP and IP, mTOR is a well-known inhibitor of ATG, which is also involved in proteostasis, neurotransmission, and neuro-immunity (146, 147). In the last decades, evidence emerged indicating an intimate biochemical and morphological interplay between UP and ATG (107, 148, 149). In fact, UP and ATG-lysosomal pathway can be simultaneously modulated to prevent or slow down the disease process, as shown in experimental models (102, 148, 149). Recent studies showed that ATG-like vacuoles of choroid plexus epithelial cells release active UP subunits in the CSF (150). Since choroid cells express IP and MHC-I molecules to act as APCs, it is likely that IP is strategically placed at this level to modulate neuro-immunity during T-cell trafficking to the brain. The IP is also strategically placed within microvascular endothelial cells (151, 152). Here, the IP may modulate the luminal expression of MHC-I-bound CNS-derived Ags, which may preferentially drive the recruitment of CD8+ effector T-cells to the brain parenchyma (24). These findings open novel avenues to experimental studies aimed at dissecting the role of UP and the interplay with ATG in the context of neuro-immune pathophysiology.

## Author Contributions

FL wrote the article and made artwork. FB contributed to conceptualization. AG and CLB contributed to the literature review and artwork. FF coordinator of the paper, he drafted the article and critically revised the article for important intellectual content.

### Conflict of Interest Statement

The authors declare that the research was conducted in the absence of any commercial or financial relationships that could be construed as a potential conflict of interest.
